# Establishing causal relationships between sleep and adiposity traits using Mendelian randomization

**DOI:** 10.1002/oby.23668

**Published:** 2023-02-15

**Authors:** Bryony L. Hayes, Marina Vabistsevits, Richard M. Martin, Deborah A. Lawlor, Rebecca C. Richmond, Timothy Robinson

**Affiliations:** ^1^ Medical Research Council Integrative Epidemiology Unit University of Bristol Bristol UK; ^2^ Population Health Sciences, Bristol Medical School University of Bristol Bristol UK; ^3^ NIHR Bristol Biomedical Research Centre University Hospitals Bristol and Weston National Health Services Foundation Trust and the University of Bristol Bristol UK; ^4^ Bristol Cancer Institute University Hospitals Bristol and Weston National Health Services Foundation Trust Bristol UK

## Abstract

**Objective:**

The aim of this study was to systematically evaluate the direction of any potential causal effect between sleep and adiposity traits.

**Methods:**

Two‐sample Mendelian randomization was used to assess the association of genetically predicted sleep traits with adiposity and vice versa. Using data from UK Biobank and 23andMe, the sleep traits explored were morning preference (chronotype; *N* = 697,828), insomnia (*N* = 1,331,010), sleep duration (*N* = 446,118), napping (*N* = 452,633), and daytime sleepiness (*N* = 452,071). Using data from the Genetic Investigation of ANthropometric Traits (GIANT) and Early Growth Genetics (EGG) consortia, the adiposity traits explored were adult BMI, hip circumference (HC), waist circumference (WC), waist‐hip ratio (WHR; *N* = 322,154), and childhood BMI (*N* = 35,668).

**Results:**

This study found evidence that insomnia symptoms increased mean WC, BMI, and WHR (difference in means, WC = 0.39 SD [95% CI: 0.13‐0.64], BMI = 0.47 SD [95% CI: 0.22‐0.73], and WHR = 0.34 SD [95% CI: 0.16‐0.52]). Napping increased mean WHR (0.23 SD [95% CI: 0.08‐0.39]). Higher HC, WC, and adult BMI increased odds of daytime sleepiness (HC = 0.02 SD [95% CI: 0.01‐0.04], WC = 0.04 SD [95% CI: 0.01‐0.06], and BMI 0.02 SD [95% CI: 0.00‐0.04]). This study also found that higher mean childhood BMI resulted in lower odds of napping (−0.01 SD [95% CI: 0.02‐0.00]).

**Conclusions:**

The effects of insomnia on adiposity and of adiposity on daytime sleepiness suggest that poor sleep and weight gain may contribute to a feedback loop that could be detrimental to overall health.

**Study Importance**

**What is already known?**

- Higher adult BMI increases daytime napping, daytime sleepiness, and morning preference, and more‐frequent insomnia symptoms increase adult BMI.
- Greater waist circumference (WC) and waist‐hip ratio (WHR) increase daytime napping.
- Longer sleep duration reduces childhood BMI, and more‐frequent napping may increase WC and WHR.

**What does this study add?**

- This study provides evidence that higher mean childhood BMI results in less‐frequent napping as an adult.
- This study provides evidence that more‐frequent insomnia symptoms increase mean WC and WHR, with little evidence for an effect in the opposing direction of adiposity on insomnia.
- This study provides consistent findings with previously reported results, supported by additional sensitivity analyses.

**How might these results change the direction of research or the focus of clinical practice?**

- Our results show that experiencing more‐frequent insomnia symptoms increases BMI and other adiposity traits. Therefore, someone who suffers from insomnia may struggle to lose weight without first dealing with the insomnia.
- A better understanding of the relationship between sleep and adiposity traits may help individuals who struggle to maintain healthy sleep or healthy weight, improve overall health, and consequently reduce the economic burden on our health care system.

## INTRODUCTION

Poor sleep is common, with up to 67% of UK adults reporting disturbed sleep, 26% to 36% experiencing insomnia, and 23% sleeping for <5 hours per night [1]. Sleep traits such as chronotype (i.e., morning or evening preference), insomnia, and sleep duration have previously been studied in relation to having both overweight and obesity. Sleep disorders and obesity have been linked to almost every aspect of health, from mental health [2, 3, 4] to overall physical health [4, 5, 6, 7, 8, 9]. Therefore, establishing the extent to which they relate to each other is important for identifying modifiable targets for interventions that could have beneficial effects on healthy sleep and weight and thereby other health outcomes.

Conventional multivariable regression analyses show reported evening preference, insomnia, and short and long sleep duration to associate with increased odds of obesity (body mass index [BMI] ≥ 30 kg/m^2^) [10, 11]. Studies such as these have predominantly explored whether sleep has an impact on adiposity, with few investigating whether there is a reverse relationship, i.e., a potential effect of adiposity on sleep traits. Understanding whether there is a bidirectional effect is important in itself, as well as for understanding whether associations of sleep traits with adiposity are biased by reverse causation. Furthermore, using conventional regression methods, it is difficult to determine whether these associations are causal or explained by residual confounding. However, the Mendelian randomization (MR) approach applied in this study is able to circumvent these issues.

MR is a causal inference approach that uses germline genetic variants associated with potentially modifiable risk factors as instruments to estimate causal effects on outcomes. MR is less vulnerable to biases incurred by conventional observational analyses, such as reverse causation and confounding, although there are a set of assumptions that can produce biased estimates when violated [12, 13, 14].

We have identified three existing MR studies that have explored potential causal effects between adiposity and sleep traits (Table 1) [15, 16, 17]. Together, these suggest that higher adult BMI (adult‐BMI) potentially increases daytime napping, daytime sleepiness, and morning preference; greater waist circumference (WC) and waist‐hip ratio (WHR) increase daytime napping; longer sleep duration reduces childhood BMI (child‐BMI); and more‐frequent napping may increase WC and WHR. None of these studies systematically explored a range of sleep traits with a range of adiposity traits within the same study, making it difficult to establish potential bidirectional effects from these separate studies, and most did not undertake sensitivity analyses to explore bias due to assumption violations.

**TABLE 1:** Summary of previously published two‐sample MR studies for sleep and adiposity traits

Study	Exposure	Outcome	Sample size (outcome)	Sensitivity analyses	β (95% CI)	*p* value
**Dashti et al., 2021 [** **15** **]**	Sleep duration	BMI	339,224	No	0.039 (−0.06 to 0.13)	4.20 E‐01
	Daytime napping	BMI	339,224		0.21 (0.02 to 0.40)	3.00 E‐02
	Morning preference	BMI	339,224		−0.01 (−0.05 to 0.03)	5.90 E‐01
	Daytime sleepiness	BMI	339,224		0.20 (−0.33 to 0.73)	4.60 E‐01
	Insomnia	BMI	339,224		0.36 (0.26 to 0.46)	1.25 E‐12
	BMI	Sleep duration	446, 118		0.01 (−0.04 to 0.07)	6.30 E‐01
	BMI	Daytime napping	452,633		0.03 (0.00 to 0.05)	4.00 E‐02
	BMI	Morning preference	697,828		0.06 (0.00 to 0.12)	4.60 E‐02
	BMI	Daytime sleepiness	452,071		0.02 (0.01 to 0.03)	4.00 E‐04
	BMI	Insomnia	1,331,010		0.01 (−0.01 to 0.03)	4.20 E‐01
**Dashti et al., 2021 [** **16** **]**	Daytime napping	WC	232,101	Limited	0.28 (0.11 to 0.45)	1.00 E‐03
	Insomnia	WHR	210,082		0.29 (0.17 to 0.41)	1.58 E‐07
	Sleep duration	Obesity (clinically determined)	16,033		1.00 (0.99 to 1.00)	2.40 E‐01
	WC	Daytime napping	452,633		0.03 (−0.01 to 0.07)	1.10 E‐01
	WHR	Insomnia	1,331,010		−0.03 (−0.05 to −0.01)	7.16 E‐07
	WHR (adj. BMI)	Sleep duration	446,118		−0.14 (−0.19 to −0.08)	5.03 E‐06
**Wang et al., 2019 [** **17** **]**	Sleep duration	Child‐BMI	35,668	Limited	−0.29 (−0.54 to −0.04)	2.20 E‐02

Abbreviation: adj., adjusted; child‐BMI, childhood BMI; MR, Mendelian randomization; WC, waist circumference; WHR, waist‐hip ratio.

Our aim was to systematically evaluate the potential causal direction of effect between sleep and adiposity traits.

## METHODS

We used two‐sample MR analyses in which the associations of the germline genetic instrumental variants (IVs) with both the exposure (sample 1) and the outcome (sample 2) were derived from two independent (i.e., nonoverlapping) samples. Sleep traits explored in this study were as follows: morning preference; insomnia; sleep duration; napping during the day; and daytime sleepiness. Adiposity traits explored in this study included adult‐BMI, child‐BMI, WC, hip circumference (HC), and WHR.

**FIGURE 1:**
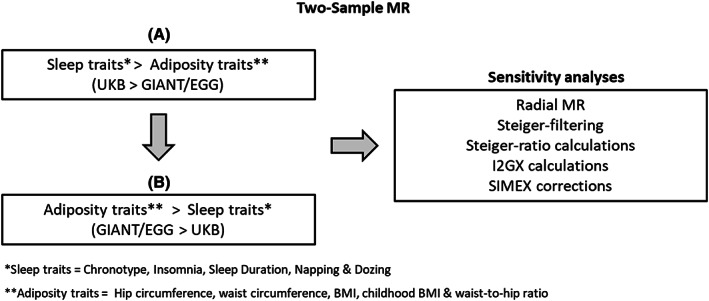
Study design. Two‐sample MR is used in the main analyses to explore the relationship between sleep and adiposity, and sensitivity analyses are used to assess robustness of these associations. EGG, Early Growth Genetics; GIANT, Genetic Investigation of ANthropometric Traits; MR, Mendelian randomization; SIMEX, simulation extrapolation method; UKB, UK Biobank

Further information regarding study design is provided in Figure 1.

### Genetic instruments for sleep traits

Genetic instruments for sleep duration, napping, and daytime dozing traits used in two‐sample MR were generated from genome‐wide association studies (GWAS) conducted in UK Biobank (UKB) [18, 19], and instruments for morning preference and insomnia were generated from GWAS conducted in a meta‐analysis of UKB and 23andMe. All sleep traits explored in this study were self‐reported, and all participants were of self‐reported European ancestry. For this, a linear mixed‐model association method was used to account for relatedness and population stratification with BOLT‐LMM software (version 2.3) [20]. At baseline, participants completed a touch screen questionnaire, which included questions about their sleep behaviors. Details of these questions are available in online Supporting Information Methods.

We identified genetic instruments for chronotype from UKB and 23andMe meta‐summary data (*N* = 697,828) [21], from which 351 independent single‐nucleotide polymorphisms (SNPs; defined as *R* ^2^ < 0.001) reached genome‐wide significance (*P* < 5 × 10^−08^). For insomnia, we identified 246 independent SNPs that reached genome‐wide significance from UKB and 23andMe meta‐summary data (*N* = 1,331,010) [22]. For sleep duration (*N* = 446,118) [23], napping (*N* = 452,633) [16], and daytime sleepiness (*N* = 452,071) [24], genetic instruments were from UKB summary data, from which 78, 93, and 38 independent SNPs that achieved genome‐wide significance were identified, respectively. Supporting Information Table S1 provides summary statistics of the IVs used to instrument each trait.

### Genetic instruments for adiposity traits

Genetic instruments for all adult adiposity traits used in two‐sample MR were generated from the Genetic Investigation of ANthropometric Traits (GIANT) consortia, a meta‐analysis of ~59 studies from across the UK and Europe [25], with those for child‐BMI generated from the Early Growth Genetics (EGG) consortia, a meta‐analysis of ~20 studies from across the UK and Europe [26]. All data used in this study were from participants of self‐reported European ancestry. BMI was calculated from weight (kilograms) divided by the square of height in meters. An adult is classified as having overweight if their BMI is 25.0 to 29.9 and as having obesity if their BMI is >30. Measures of HC and WC were both taken in centimeters, and WHR was calculated from WC divided by HC.

We identified genetic instruments for HC, WC, adult‐BMI, and WHR from GIANT consortium summary data (*N* = 322,154) [25, 27], for which 52, 41, 68, and 29 independent (defined as *R* ^2^ < 0.001) SNPs reached genome‐wide significance (*P* < 5 × 10^−08^), respectively. Genetic instruments for child‐BMI were obtained from EGG consortium summary data (*N* = 35,668) [26], for which six SNPs reached genome‐wide significance. Supporting Information Table S2 provides summary statistics of the IVs used to instrument each trait.

### Statistical analyses

The two‐sample MR approach uses genome‐wide significant IVs to obtain estimates for the causal effect of risk factors on our chosen outcomes. For the univariable MR analyses in this study, the TwoSampleMR R package was used to combine and harmonize genetic summary data for each of our sleep exposure traits to determine the causal effect on adiposity and, subsequently, for each of our adiposity traits to determine the causal effect on sleep. For all main analyses, an inverse variance‐weighted (IVW) approach was used, whereby a combined estimate of the causal effect from individual variants is obtained from the slope of a regression line through the weighted IV‐mean exposure versus IV‐mean outcome associations, with the line constrained to have an intercept of zero.

The following three key assumptions must be fulfilled to ensure the validity of an MR study for making causal inference: 1) the relevance assumption, i.e., that genetic IVs are statistically and robustly associated with the exposure of interest in the population to which inference is made; 2) the independence assumption, i.e., that there is no confounding between the genetic IVs and outcome; and 3) the exclusion restriction assumption, i.e., that genetic IVs only influence an outcome through the exposure of interest [14].

We explored instrument strength with the *F*‐statistics of the association between the IVs and each exposure 28, 29]. Population substructure can confound genetic instrument‐outcome associations; therefore, it was minimized by restricting analyses to European ancestry participants and using GWAS data that had adjusted for principal components reflecting different ancestral subpopulations. To explore the potential for unbalanced horizontal pleiotropy, we conducted sensitivity analyses using MR‐Egger [30], weighted‐median [31], and weighted‐mode [32] MR and assessed between‐SNP heterogeneity using Cochran *Q* and leave‐one‐out analyses [13, 33]. *I* ^2^ statistics were used to estimate the proportion of the variance between IV estimates that is due to heterogeneity [34]. Weighted and unweighted *I* ^2^_GX_ statistics were calculated to provide an indicator for the expected relative bias of the MR‐Egger causal estimate [35], and simulation‐extrapolation method (SIMEX) corrections were conducted to extrapolate bias‐adjusted inference where necessary [36]. To identify IVs with the largest contribution toward heterogeneity, radial MR was conducted (*α* = 0.05/nSNP) [37]. To identify instrumental SNPs more strongly associated with the outcome of interest than the exposure, Steiger filtering was conducted [38]. Following both radial MR and Steiger filtering, MR was then repeated with any outliers removed to assess their impact.

Sample overlap between UKB and GIANT/EGG is negligible; therefore, analyses between these consortia should not violate the independence assumption in two‐sample MR.

MR analyses used the R package “TwoSampleMR” (version 0.5.6) [26], R version 4.0.4.

This study was conducted in accordance with Strengthening the Reporting of Observational Studies in Epidemiology (STROBE) guidelines [39].

## RESULTS

**FIGURE 2:**
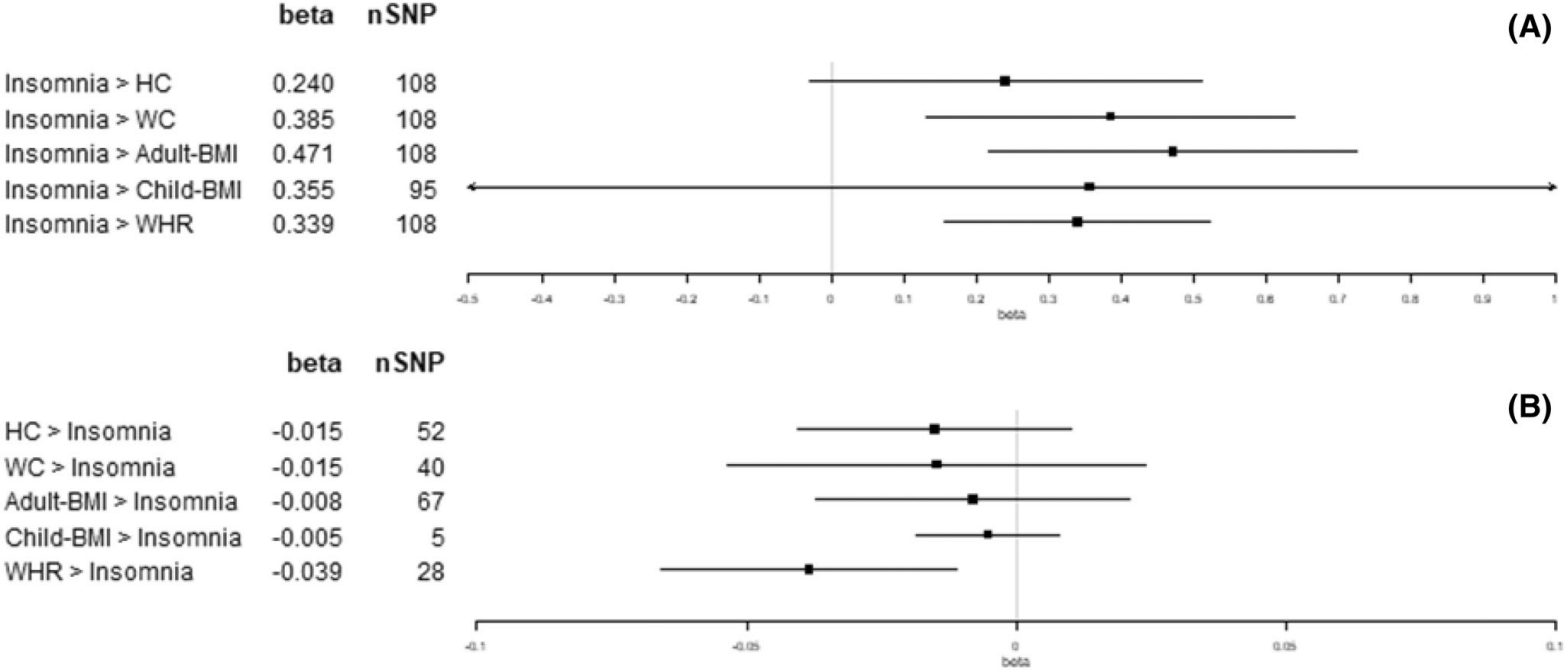
Two‐sample bidirectional Mendelian randomization. Forest plot of (A) insomnia trait effects on adiposity and (B) adiposity trait effects on insomnia. All results presented are inverse variance‐weighted. HC, hip circumference; SNP, single‐nucleotide polymorphism; WC, waist circumference; WHR, waist‐hip ratio

**FIGURE 3:**
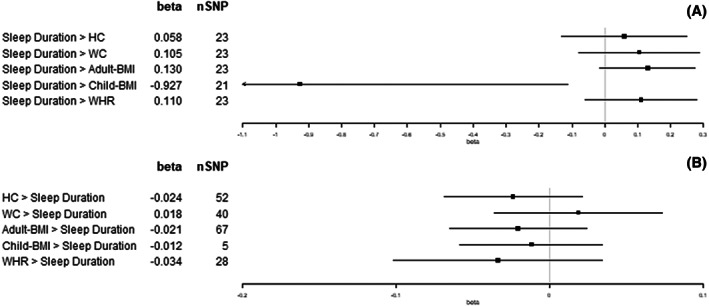
Two‐sample bidirectional Mendelian randomization. Forest plot of (A) sleep duration trait effects on adiposity and (B) adiposity trait effects on sleep duration. All results presented are inverse variance‐weighted. HC, hip circumference; SNP, single‐nucleotide polymorphism; WC, waist circumference; WHR, waist‐hip ratio

**FIGURE 4:**
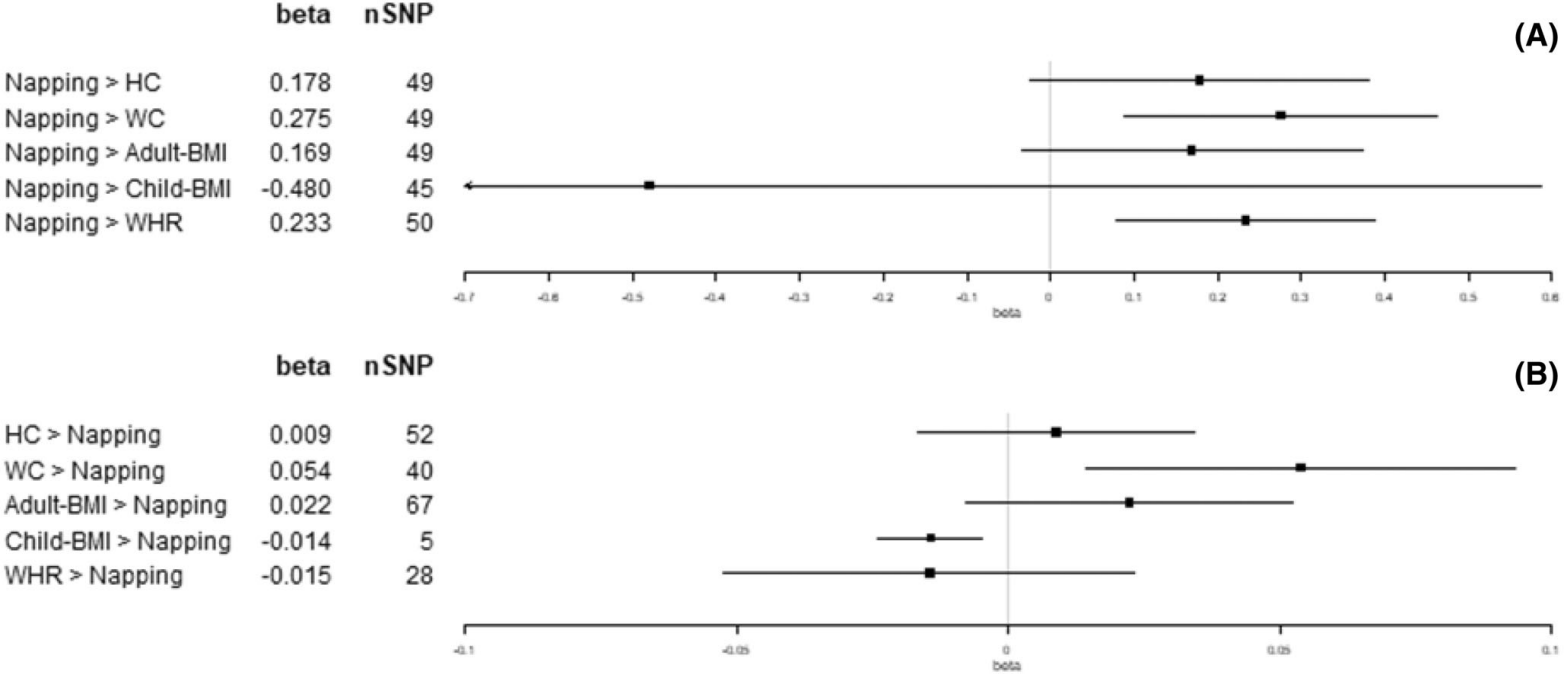
Two‐sample bidirectional Mendelian randomization. Forest plot of (A) napping trait effects on adiposity and (B) adiposity trait effects on napping. All results presented are inverse variance‐weighted. HC, hip circumference; SNP, single‐nucleotide polymorphism; WC, waist circumference; WHR, waist‐hip ratio

**FIGURE 5:**
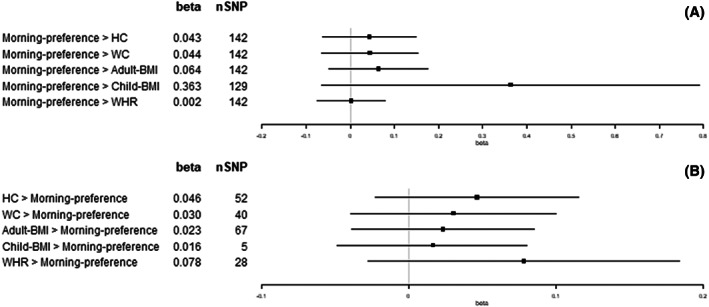
Two‐sample bidirectional Mendelian randomization. Forest plot of (A) morning preference trait effects on adiposity and (B) adiposity trait effects on morning preference. All results presented are inverse variance‐weighted. HC, hip circumference; SNP, single‐nucleotide polymorphism; WC, waist circumference; WHR, waist‐hip ratio

**FIGURE 6:**
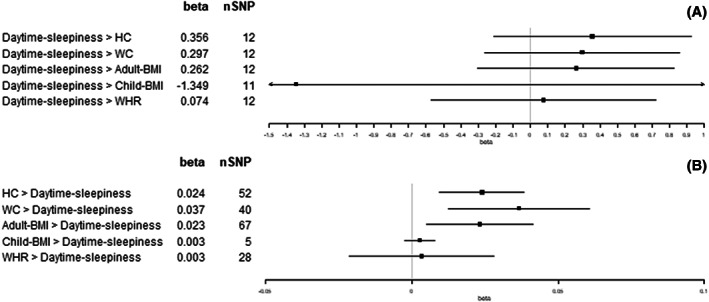
Two‐sample bidirectional Mendelian randomization. Forest plot of (A) daytime sleepiness trait effects on adiposity and (B) adiposity trait effects on daytime sleepiness. All results presented are inverse variance‐weighted. HC, hip circumference; SNP, single‐nucleotide polymorphism; WC, waist circumference; WHR, waist‐hip ratio

We observed an increase in WC (1 standard deviation [SD] = 12.5 cm), adult‐BMI (5.1 kg/m^2^), and WHR (1 SD = 0.07) for each category increase in insomnia symptoms (from never/rarely, to sometimes, and to usually; β = 0.39 SD, 95% confidence interval [CI]: 0.13 to 0.64; β = 0.47 SD, 95% CI: 0.22 to 0.73; and β = 0.34 SD, 95% CI: 0.16 to 0.52, respectively; Figure 2). For each hour increase in sleep duration, we observed a decrease in child‐BMI (SD = 4.7 kg/m^2^; β = −0.93 SD, 95% CI: −1.74 to −0.11; Figure 3). For each category increase in napping, we observed an increase in WC and WHR (β = 0.28 SD, 95% CI: 0.09 to 0.46; and β = 0.23 SD, 95% CI: 0.08 to 0.39, respectively; Figure 4). There was little evidence for effects of sleep traits on adiposity traits (Figures 2, 3, 4, 5, 6). For all analyses, results from MR‐Egger, weighted‐median, and weighted‐mode methods were similar to IVW estimates (Supporting Information Table S3).

Results from most sensitivity analyses for effects of different sleep traits on adiposity were consistent with the main analysis results. With the exception of sleep duration effect on child‐BMI, in which the results were attenuated following Steiger filtering (β = −1.77 SD, 95% CI: −7.24 to 3.39), they remained directionally consistent with those reported in the main analysis (Supporting Information Table S4).

*R* ^2^ values suggested that the instruments explained 0.13% to 2.07% of the variance of the exposures, and genetic variants contributing to the sleep trait instruments had a combined *F*‐statistic of 13.2 to 48.4, indicating reasonable instrument strength [28, 40]. Between‐IV heterogeneity for sleep trait instruments ranged from 0% to 88% (*Q*_stat_ = 19‐1126; *Q*_pval_ = 3.6 × 10^−153^ to 4.8 × 10^−1^). Weighted and unweighted *I* ^2^_GX_ [35] was also calculated for each of the sleep traits on adiposity traits and found to be between 0% and 74%. SIMEX corrections [36] were conducted for each analysis to account for this and were found to be largely consistent with MR‐Egger results reported in the main analyses (Supporting Information Table S3).

Per SD increase in HC (1 SD = 9.2 cm), we observed a category increase in daytime sleepiness (β = 0.02 SD, 95% CI: 0.01 to 0.04; Figure 6). Per SD increase in WC (1 SD = 12.5 cm), we observed a category increase in both napping and daytime sleepiness (β = 0.05 SD, 95% CI: 0.01 to 0.09; and β = 0.04 SD, 95% CI: 0.01 to 0.06, respectively; Figures 4 and 6). Per SD increase in adult‐BMI (1 SD = 5.1 kg/m^2^), we observed a category increase in daytime sleepiness (β = 0.02 SD, 95% CI: 0.00 to 0.04; Figure 6). Per SD increase in child‐BMI (1 SD = 4.7 kg/m^2^), we observed a category decrease in napping (β = −0.01 SD, 95% CI: −0.02 to 0.00; Figure 4). Per SD increase in WHR (1 SD = 0.07), we observed a category decrease in insomnia symptoms (β = −0.04 SD, 95% CI: −0.07 to −0.01; Figure 2). There was no other evidence found for effects of adiposity traits on sleep (Figures 2, 3, 4, 5, 6). For all analyses, results from MR‐Egger, weighted‐median, and weighted‐mode methods were similar to IVW estimates (Supporting Information Table S5).

Results from most sensitivity analyses for effects of different adiposity traits on sleep were consistent with the main analysis results (Supporting Information Table S6).

Genetic variants contributing to the adiposity trait instruments had a combined *F*‐statistic of 43.2 to 68.2, indicating good instrument strength [28, 40], and *r* ^2^ values suggested that instruments explained 0.98% to 2.00% of the variance of the exposures. Between‐IV heterogeneity for adiposity trait instruments ranged from 0% to 97% (*Q*_stat_ = 3‐399; *Q*_pval_ = 3.8 × 10^−49^ to 3.6 × 10^−1^). Weighted and unweighted *I* ^2^_GX_ [35] was also calculated for each of the adiposity traits on sleep traits and found to be between 0% and 91%. SIMEX corrections [36] were conducted for each analysis to account for this and found to be largely consistent with MR‐Egger results reported in the main analyses (Supporting Information Table S5).

## DISCUSSION

### Summary of main findings

This study assessed the direction of effect between a series of adiposity and sleep traits using two‐sample MR analyses. Overall, we found consistent MR evidence, including from radial MR and Steiger filtering, of insomnia symptoms increasing mean WC, BMI, and WHR, with little evidence for an effect in the opposing direction of adiposity on insomnia. There was evidence that napping increased mean WHR, but no effect was found in the other direction. Our results suggest higher mean child‐BMI results in lower odds of napping, and that longer sleep duration may result in lower child‐BMI, although, for the latter, Steiger filtering suggested the presence of shared causal variants more strongly associated with child‐BMI, and there was no evidence for an effect in the opposing direction of adiposity on sleep duration.

A bidirectional adverse effect was found between napping and WC, which was consistent across radial MR and Steiger‐filtered results. We found little evidence for an effect of daytime sleepiness on adiposity in our main results; however, evidence for an adverse effect of daytime sleepiness on HC, WC, and BMI was found in radial MR (and on WC and BMI in Steiger‐filtered results). Reciprocal adverse effects were found in the opposing direction for the effect of HC, WC, and BMI on daytime sleepiness, which was consistent across radial MR and Steiger‐filtered results.

### Public health and clinical implications

The public health and clinical implications of these results are potentially far‐reaching. Our results show that experiencing more‐frequent insomnia symptoms increases BMI (Figure 2A). Therefore, someone who suffers from insomnia may struggle to lose weight without first dealing with their insomnia.

Overall, a better understanding of the complex relationship between sleep and adiposity traits may help individuals who struggle to maintain healthy sleep or healthy weight, improve overall health, and consequently reduce the economic burden on our health care system.

### Comparison with previous literature

The effects of insomnia on adult‐BMI (1 SD = 5.1 kg/m^2^) and WHR (1 SD = 0.07) found in this study (β = 0.47 SD, 95% CI: 0.22‐0.73; and β = 0.34 SD, 95% CI: 0.16‐0.52, respectively) are consistent with but less conservative than previously reported two‐sample MR findings by Xiuyan et al. (β = 0.08 SD, 95% CI: 0.06‐0.09; and β = 0.03 SD, 95% CI: 0.02‐0.04, respectively) [41]. This may be attributed to the different GWAS used for their insomnia exposure (a meta‐GWAS of UKB and 23andMe vs. UKB only) as well as differences in underlying sample population demographics. Our study only used participants of European descent, whereas Xiuyun et al. used a “mixed” population for their WHR GWAS. Another study by Dashti et al. also explored the effects of insomnia on BMI (1 SD = 5.1 kg/m^2^), of which the results were consistent with those reported here (β = 0.36 SD, 95% CI: 0.26‐0.46) [15].

The effect of sleep duration on child‐BMI found in this study (β = −0.93 SD, 95% CI: −1.74 to −0.11) is consistent with but less conservative than the previous reported two‐sample MR findings (1 SD = 4.65 kg/m^2^; β = −0.27 SD, 95% CI: −0.51 to −0.02) [17]. The same study by Wang et al. also tested the robustness of this effect in supplementary analyses by correction with MR‐Pleiotropy RESidual Sum and Outlier (PRESSO) and found consistent results (β = −0.31, 95% CI: −0.53 to −0.01). In contrast, our study reported some attenuation of effect following removal of SNPs more strongly associated with the outcome in Steiger filtering (β = −0.65 SD, 95% CI: −1.56 to 0.25). We have also taken note of the imprecisely estimated MR‐Egger, median and mode results, and the weighted and unweighted *I* ^2^_GX_ result of 0% in our main analyses, suggesting a large amount of measurement error bias (Supporting Information Table S3) [35].

The bidirectional effect between napping and WC found in this study (β = 0.28 SD, 95% CI: 0.09‐0.46 vs. β = 0.05 SD, 95% CI: 0.01‐0.09) is consistent with the previously reported two‐sample MR findings by Dashti et al. (β = 0.28 SD, 95% CI: 0.11‐0.45 vs. β = 0.03 SD, 95% CI: 0.01‐0.07) [16]. The unidirectional effect we found for napping on WHR (β = 0.23 SD, 95% CI: 0.08‐0.39) is also consistent with the previously reported results (β = 0.19 SD, 95% CI: 0.04‐0.33), which is to be expected given the same underlying data used for these analyses (UKB and GIANT summary statistics) [16]. Furthermore, our additional sensitivity analyses with radial MR and Steiger filtering found these associations to be robust.

To our knowledge, the unidirectional effect of child‐BMI on napping found in this study (β = 0.28 SD, 95% CI: 0.09‐0.46) has not previously been reported. No SNPs were flagged for removal in either radial MR or Steiger filtering for this analysis, but weighted and unweighted *I* ^2^_GX_ was found to be 0% to 14%, suggesting a large amount of measurement error bias (Supporting Information Table S4) [35].

The unidirectional effect of adult‐BMI on daytime sleepiness reported in the main results of this study (β = 0.02 SD, 95% CI: 0.00‐0.04) is consistent with that previously found by Dashti et al. (β = 0.02 SD, 95% CI: 0.01‐0.03) [15]; furthermore, this result persists after removal of outliers in radial MR (β = 0.02 SD, 95% CI: 0.01‐0.04). HC and WC were also found to increase daytime sleepiness (β = 0.02 SD, 95% CI: 0.01‐0.04; and β = 0.04 SD, 95% CI: 0.01‐0.06, respectively), both of which were robust to radial MR and Steiger filtering analyses. Although our main results in the opposing direction report little effect of daytime sleepiness on adult‐BMI, HC, or WC, this may be attributed to the moderate‐to‐high heterogeneity between SNPs for this instrument (*I* ^2^ = 59% to 73%), leading to imprecise estimation. Following the removal of one outlier SNP (rs6741951) from the daytime sleepiness instrument in radial MR, heterogeneity between SNPs was reduced to 0% to 21%, and an adverse effect was then found for adult‐BMI (β = 0.48 SD, 95% CI: 0.13‐0.82), HC (β = 0.55 SD, 95% CI: 0.12‐0.97), and WC (β = 0.51 SD, 95% CI: 0.14‐0.88). Altogether, the evidence suggests that a bidirectional relationship may exist between daytime sleepiness and adult‐BMI, WC, and HC.

### Strengths and limitations

A key strength of this study is the use of two‐sample MR to systematically appraise the causal effects of each of our sleep traits on adiposity and vice versa. Furthermore, MR assumptions were thoroughly tested with the use of additional sensitivity analyses such as radial MR and Steiger filtering, the results of which provide evidence for the robustness of our results. The genetic summary data used for all traits in this study were obtained from the largest available GWAS while still maintaining zero overlap between exposure and outcome data sets.

Although we were thorough in our assessment of MR assumptions using various sensitivity analyses, we were not able to directly appraise independence of IVs from potential confounding factors. Given that MR uses germline IVs, it is largely understood that these will not be influenced by confounders. Minimizing population stratification may help to alleviate concerns of independence assumption violation [42], but this is difficult to test in a two‐sample MR framework.

The use of overlapping sample populations between exposures and outcomes in a two‐sample MR setting may be a potential source of bias [43]. Therefore, despite the availability of GWAS with larger sample sizes generated from meta‐analysis of UKB and either GIANT or EGG data, we opted to use GWAS that used UKB‐only sample populations for our sleep traits and GIANT‐only or EGG‐only sample populations for our adiposity traits to ensure zero sample overlap between exposure and outcomes for our analyses.

The use of self‐reported measures of sleep may be another potential source of information bias. However, all sleep traits investigated in this study have been previously validated in independent data sets and/or with the use of comparable objective measures from accelerometer data in UKB. For example, morning preference chronotype is strongly correlated (rg = 0.903) with objectively measured least active 5 hours (L5‐timing) [16, 21, 22, 23, 24].

### Further work

The analyses presented here demonstrate robust casual evidence for both unidirectional and bidirectional relationships between sleep and adiposity; therefore, further investigation is required to inform clinical guidelines and policy. To improve the robustness of the findings in this study, it would be interesting to investigate the associations found using polygenic risk scores, in which multiple genetic IVs are combined into a single univariate score, as well as objective measures that correspond to self‐report sleep traits, such as accelerometer‐derived sleep duration versus self‐report sleep duration [44]. It may also be of interest to conduct further studies using additional measures of adiposity such as waist to height ratio should appropriate GWAS data become available. Furthermore, genetic epidemiological studies are disproportionately conducted in population samples of European ancestry. Therefore, future studies that include populations from a variety of ancestries will only serve to better our understanding of the genetics that underpin these associations.

In this study, directionality was explored between sleep and adiposity. Moving forward, it would be interesting to use these results to inform and conduct mediation analyses to look at effects on outcomes such as cancers and cardiovascular disorders.

## CONCLUSION

This study has extended previous findings regarding the effect of sleep on adiposity and vice versa and provided robust evidence for these associations across a variety of methods. Collectively, the effect of insomnia on adiposity and adiposity on daytime sleepiness suggests that poor sleep and weight gain may contribute to a feedback loop that is detrimental to the overall health of the individual. Further understanding of these interactions and how they together might impact disease outcomes would be highly beneficial and informative for intervention studies seeking to improve overall health and consequently reduce the economic burden on our health care system.

## FUNDING INFORMATION

Bryony L. Hayes is funded by an Above & Beyond breast cancer legacy grant from University Hospitals Bristol National Health Service (NHS) Foundation Trust (www.aboveandbeyond.org.uk). Marina Vabistsevits is supported by the University of Bristol Alumni Fund (Professor Sir Eric Thomas Scholarship). Timothy Robinson is supported by a National Institute for Health Research (NIHR) Development and Skills Enhancement Award (NIHR302363). Rebecca C. Richmond is a de Pass Vice Chancellor's Research Fellow. Richard M. Martin is supported by a Cancer Research UK (C18281/A19169) programme grant (the Integrative Cancer Epidemiology Programme; www.cancerresearchuk.org/funding-for-researchers). Bryony L. Hayes, Timothy Robinson, Richard M. Martin, Deborah A. Lawlor, and Rebecca C. Richmond work in the Medical Research Council Integrative Epidemiology Unit at the University of Bristol, supported by the Medical Research Council (MC_UU_00011/1 and MC_UU_00011/6 (www.mrc.ukri.org) and the University of Bristol. Richard M. Martin and Deborah A. Lawlor are also supported by the NIHR Bristol Biomedical Research Centre, which is funded by the NIHR (www.nihr.ac.uk) and is a partnership between University Hospitals Bristol and Weston NHS Foundation Trust and the University of Bristol. Deborah A. Lawlor is an NIHR Senior Investigator (NF‐0616‐10102) and is supported by the British Heart Foundation (CH/F/20/90003 and AA/18/7/34219) and Diabetes UK (17/0005700). The funders had no role in study design, data collection and analysis, decision to publish, or preparation of the manuscript.

## CONFLICT OF INTEREST

Deborah A. Lawlor has received support from Roche Diagnostics and Medtronic plc for research unrelated to that presented here. Timothy Robinson has received grants from Daiichi‐Sankyo Company, Limited and Amgen Inc. to attend educational workshops. All other authors declared no conflict of interest.

## Supporting information


**TABLE S1.** Exposures used in MR analyses. MR, Mendelian randomization.
**TABLE S2.** Exposures used in MR analyses. MR, Mendelian randomization.
**TABLE S3.** Sleep trait effects on adiposity using two‐sample MR. MR, Mendelian randomization.
**TABLE S4.** Sleep trait effects on adiposity using two‐sample MR with outliers removed by radial MR or Steiger filtering. MR, Mendelian randomization.
**TABLE S5.** Adiposity effects on sleep traits using two‐sample MR. MR, Mendelian randomization.
**TABLE S6.** Adiposity effects on sleep traits using two‐sample MR with outliers removed by radial MR or Steiger filtering. MR, Mendelian randomization.

**DATA S1.** Supporting Information

## Data Availability

This research was conducted using the UK Biobank Resource under application number 16391. Summary data for the morning preference, insomnia, sleep duration, napping, and daytime sleepiness genome‐wide association studies (GWAS) used in this study are available at the following link: https://sleep.hugeamp.org/dinspector.html?dataset=GWAS_UKBB_eu Summary data for the adult BMI, waist circumference, hip circumference, and waist‐hip ratio GWAS used in this study are available at the following link: https://portals.broadinstitute.org/collaboration/giant/index.php/GIANT_consortium_data_files Summary data for the child BMI GWAS used in this study is available at the following link: http://egg-consortium.org/childhood-bmi.html
